# Multivessel coronary artery disease, free fatty acids, oxidized LDL and its antibody in myocardial infarction

**DOI:** 10.1186/1476-511X-13-111

**Published:** 2014-07-09

**Authors:** Olga Gruzdeva, Evgenya Uchasova, Yulia Dyleva, Ekaterina Belik, Victoria Karetnikova, Alexander Shilov, Olga Barbarash

**Affiliations:** 1 Research Institute for Complex Issues of Cardiovascular Diseases under the Siberian Branch of the Russian Academy of Medical Sciences, Kemerovo, Russia

**Keywords:** Myocardial infarction, Free fatty acid, Oxidized LDL, Antibodies

## Abstract

**Background:**

Free fatty acids (FFA), oxidized low-density lipoprotein (LDL) and its antibodies, lipid profile markers, which are formed under oxidative stress, play an important role in atherosclerotic disease. Assess the levels of these markers in myocardial infarction patients depending on the extent of coronary artery disease (CAD).

**Methods:**

ST-elevation MI patients with hemodynamically significant stenoses of ≥75% in one, two, three, or more coronary arteries were examined. The patients were divided into three groups according to the severity of coronary lesions. Patients had a ≥75% stenotic lesion in one coronary artery (group 1, n = 135), two coronary arteries (group 2, n = 115), or three or more coronary arteries (group 3, n = 150). The control group comprised healthy subjects (n = 33).

**Results:**

FFA levels on day 1 from MI onset were higher in groups 1, 2, and 3 compared with controls. On day 1 from MI onset, oxidized LDL levels were significantly higher in groups 2 and 3 than those in controls (both р = 0.001). Oxidized LDL levels were significantly higher in patients with multivessel CAD compared with those with single-vessel CAD on days 1 and 12. Antibody levels increased with the number of affected arteries.

**Conclusion:**

High levels FFA, oxidized LDL and its antibody, lipid profile markers, and parameters of the pro/antioxidant systems persist during the subacute phase of MI.

## Introduction

Myocardial infarction (MI) in patients with coronary artery disease (CAD) of different severity remains the leading cause of cardiovascular death. Early MI diagnosis, assessment of CAD severity, and secondary event risk prediction are the most important factors for preventing mortality. A previous study showed that the incidence of significant cardiovascular events in multivessel CAD patients was 23.6% vs. 19.5% in patients with two-vessel disease and 14.5% in those with single-vessel disease [1]. The 5-year risk of death in MI patients with multivessel CAD is increased by two times compared with healthy patients [2].

Dyslipidemia, which has a significant impact on MI, is a well-established factor contributing to the risk of atherosclerosis. However, dyslipidemia does not explain all of the cases of acute coronary events. According to Ansell et al., 50% of all coronary events occur without a history of hypercholesterolemia [3]. In patients with normal high-density lipoprotein (HDL-C) levels, the number of coronary events is 30% less than that in those with decreased low-density lipoprotein (LDL-C) levels [4]. Moreover, a significant number of coronary events occur in those with normal LDL-C levels [5]. All of these factors indicate that new markers of an adverse course of CAD, especially in case of multivessel disease, are required.

Measuring blood levels of free fatty acids (FFAs) can have certain diagnostic value. FFAs carry out some important functions, including ATP production, and they act as cell signal mediators (activation of various protein kinase C isoforms and initiation of apoptosis), ligand transcription factors, and basic components of biological membranes [6]. Some authors consider that increased FFAs levels are the earliest predictor of ischemia and a more sensitive marker of the severity of ischemia than electrocardiographic studies [7]. The results of prospective and clinical trials show a strong correlation between increased plasma FFA levels, CAD, and sudden risk of death [8]. Furthermore, FFAs are regarded as potential biochemical markers of postinfarct myocardial remodeling [9]. Laboratory monitoring of blood FFA levels in acute coronary events can play an important role in choosing a treatment strategy for risk stratification in this patient category.

Measuring oxidized low-density lipoprotein (oxidized LDL), which plays an important role in atherosclerotic plaque formation and destabilization, as well as in the activation of systemic inflammation and acute coronary syndrome (ACS) development, can have diagnostic value. The level of oxidized LDL is an independent predictor of MI. In a study of 3033 patients, the risk of MI in patients with increased LDL levels was increased two-fold [10]. As a response to production of oxidized LDL, which has immunogenic potential, antibodies and immune complexes are produced, which in turn, can lead to further endothelial damage. Antibodies to oxidized LDL are supposed to play a key role in regulating oxidized LDL levels. Several studies have shown protective properties of antibodies, which may neutralize pathogenic and immunogenic activity of oxidized LDL in vivo physiological conditions and, thereby reduce the probability of atherosclerosis development. In others, their pathogenic activity is largely discussed. Elevated levels of autoantibodies to oxidized LDL may be regarded as a predictor of atherosclerosis and ACS [10,11].

Therefore, the purpose of this study was to assess the in-hospital levels of FFA in ST-elevation MI patients depending on the extent of CAD.

## Methods

### Study subjects and design

A total of 400 patients (mean [±SD] age, 60.25 ± 1.11 years) with ST-elevation MI who were admitted within 24 hours from disease onset, were enrolled in the study. MI was diagnosed according to the criteria of the 2007 Russian National Society of Cardiology Guidelines. The inclusion criteria were angina chest pain of > 20 min in duration refractory to nitroglycerin and signs of subepicardial injury. These signs included ST segment elevation on electrocardiogram, new complete left bundle branch block, and elevated cardiac enzymes, such as the MB fraction of creatine phosphokinase (CK-MB) and troponin T. The exclusion criteria were a history of type 2 diabetes mellitus, MI during percutaneous coronary intervention (PCI) or coronary artery bypass surgery, end-stage renal failure, known neoplasms, and a history of other concomitant diseases, which could significantly decrease life expectancy, including systemic and connective tissue diseases.

Maximum CK-MB levels in ST-elevation MI patients were 137.64 (121.32;167.65) U/L and maximum troponin T levels were 0.71 (0.54;0.87) ng/mL. Left ventricular ejection fraction, and end-diastolic and end-systolic volumes (EF, EDV, ESV), reflecting structural and functional myocardial remodeling during MI, were 50.42 (49.32;52.76)%, 156.43 (149.09;164.76) mL, and 75.22(70.54;80.21) mL, respectively.

The presence and severity of CAD were assessed by means of coronary angiography within the first hours from hospital admission. According to the severity of coronary lesions, the patients were divided into three groups. Stenoses of ≥ 75% were considered hemodynamically significant.

Group 1 consisted of 135 MI patients with a ≥ 75% stenotic lesion in one coronary artery. Group 2 included 115 MI patients with ≥ 75% stenoses in two coronary arteries. Group 3 consisted of 150 individuals with ≥ 75% stenoses in three or more coronary arteries. The patients of the three groups were similar in sex and age (Table 1). However, patients with two- or three-vessel CAD (groups 2 and 3) had cardiovascular risk factors such as arterial hypertension, hypercholesterolemia and smoking more frequently than patients with one-vessel CAD (group 1, Table 1). Groups 1 and 2 patients also had a history of angina pectoris, previous MI, or previous strokes/transient ischemic attacks more frequently than group 1 patients. The distribution of patients who had stage I or II chronic heart failure was similar in the groups. Stage III chronic heart failure was significantly more frequent in patients with single-vessel CAD than in patients with two- or three-vessel CAD (Table 1). Cardiogenic shock was diagnosed in the three groups with multi vessel CAD. In all of the groups, anterior Q-wave MI was predominant. However, in patients with single-vessel and three-vessel CAD, this type of infarction was observed more often than in those with two-vessel CAD. Posterior MI was diagnosed significantly more often in patients with two, three, or more affected arteries. With regard to MI complications, patients with single-vessel and three-vessel CAD had in-hospital arrhythmias more frequently, and those with three or more affected arteries had early post infarct angina and recurrent MI more frequently during their hospital stay. In-hospital treatment was administered according to the 2007 National Society of Cardiology Guidelines on acute ST-elevation MI diagnosis and treatment (Table 2). Fifty-four (49.1%) patients underwent PCI for an infarct-related artery, and if contraindicated (technically unfeasible), systemic thrombolysis with streptokinase (1.5 × 10^6^ IU) therapy was given, and seven (6.4%) patients had no reperfusion therapy. All of the patients were administered coronaroactive and antithrombotic therapy, including acetylsalicylic acid, clopidogrel, beta-blockers, and angiotensin-converting-enzyme inhibitors during the hospital stay, if not contraindicated. Antianginal drugs were administered according to standard. After discharge, patients continued therapy with the main classes of anti-ischemic agents and statins were taken by 88% of patients. Thirty-three age- and sex-matched patients with no cardiovascular disease were included in the control group. Lipid profile concentrations were measured at days 1 and 12 (at the end of the hospital stay) from MI onset.

**Table 1:** Initial clinical characteristics of patients

**Variable**	**Group 1, ≥ 75% stenosis of a coronary artery n = 135**	**Group 2, ≥ 75% stenosis 2 coronary n = 115**	**Group 3, ≥ 75% stenosis of 3 or more coronary arteries n = 150**	**Р (Fisher)**
Men, n (%)	90 (66.7)	75 (65.8)	105 (70)	Р_1,2,3_ ≥ 0.05
Age, year	50 (49;51)	58.15 (44.00;73.50)	59.43 (48.01;72.10)	Р_1_ ≥ 0.05
				Р_2_ = 0.019 Р_3_ = 0.017
**Factors risk**				
Arterial hypertension, n (%)	87 (64.4)	84 (73.7)	108 (72)	Р_1_ = 0.016 Р_2_ = 0.018 Р_3_ ≥ 0.05
Current smoking, n (%)	45 (33.3)	54 (47.4)	69 (46)	Р_1_ = 0.01
				Р_2_ = 0.015 Р_3_ ≥ 0.05
Family history of IHD, n (%)	45 (33.3)	63 (55.3)	63 (42)	Р_1,2,3_ ≥ 0.05
Hypercholesterolemia, n (%)	51 (37.8)	48 (42.1)	72 (48)	Р_1_ = 0.014 Р_2_ = 0.016, Р_3_ ≥ 0.05
CK- MB, U/L	99.64	123,64 (92.21;154.34)	137,64	Р_1_ ≥ 0.05
			(121.32;167.65)	Р_2_ = 0.016
	(72.3;118.54)			Р_3_ ≥ 0.05
Clinic angina to myocardial infarction, n (%)	63 (46.7)	69 (60.5)	84 (56)	Р_1_ = 0.014 Р_2_ = 0.013 Р_3_ ≥ 0.05
Previous myocardial infarction, n (%)	15 (15.6)	18 (18.4)	30 (22)	Р_1_ = 0.022 Р_2_ = 0.020 Р_3_ ≥ 0.05
Cerebrovascular accident/transient ischemic attack in history	12 (8.9)	6 (5.3)	18 (12)	Р_1_ ≥ 0.05
				Р_2_ = 0.012 Р_3_ = 0.010
**The depth of lesion**				
Q-waveMI	90 (66.7)	72 (63.2)	102 (68)	Р_1_ = 0.020 Р_2_ = 0.009 Р_3_ = 0.008
Non-Q-waveMI	45 (33.3)	42 (36.8)	48 (32)	Р_1,2,3_ ≥ 0.05
**Localization of MI**				
Posterior	42 (31.1)	45 (42.9)	57 (38)	Р_1_ = 0.017 Р_2_ = 0.019
				Р_3_ ≥ 0.05
Front	84 (62.2)	63 (55.3)	81 (60)	Р_1_ = 0.016
				Р_2_ ≥ 0.05
				Р_3_ = 0.015
**Acute heart failure**				
Killip I	105 (77.8)	90 (78.9)	111 (74)	Р_1,2,3_ ≥ 0.05
Killip II	21 (15.6)	18 (15.8)	27 (18)	Р_1,2,3_ ≥ 0.05
Killip III	9 (6.7)	0	3 (2)	Р_2_ = 0.015
Killip IV	0	3 (5.3)	12 (8)	Р_3_ = 0.017

**Notes:** p_1_ - the significance of differences between groups 1 and 2, (p ≤ 0.05); p_2_ - the significance of differences between groups 1 and 3, (p ≤ 0.05); p_3_ - the significance of differences between groups 2 and 3, (p ≤ 0. 05).

**Table 2:** Revascularization and drug therapy during follow-up

**Therapy, n (%)**	**All patients (n = 400)**
β-blockers	358 (89.5)
Angiotensin-convertingenzyme	328 (82.1)
Calcium channel blocker	278 (69.5)
Diuretics	168 (42)
Nitrates	350 (87.4)
Aspirin	351 (87.8)
Heparin	400 (100)
Clopidogrel	314 (78.5)
Statins	400 (100)
Thrombolysis	24 (6.1)
Percutaneous coronary intervention	196 (49.1)

### Assays

Serum levels of FFA, total cholesterol (TC), triacylglyceride (TAG), LDL, very low-density lipoprotein (VLDL), HDL, and apolipoproteins B and A1 (Apo B and Apo-A1, respectively) were measured with standard Thermo Fisher Scientific test systems (Thermo Fisher Scientific Oy, Vantaa, Finland) on a Konelab 30i biochemistry analyzer (Thermo Fisher Scientific Oy). Serum oxidized LDL levels, oxidized LDL antibodies, peroxide, protein thiol, C-peptide levels, and insulin levels were measured by ELISA with Biomedica (Waterloo, NSW, Australia) and Diagnostic Systems Laboratories (Webster, TX, USA) lab kits.

The study was carried out in compliance with the Helsinki Declaration, and its protocol was approved by the Ethical Committee of the Research Institute for Complex Issues of Cardiovascular Diseases under the Siberian Branch of the Russian Academy of Medical Sciences. All of the patients who participated in the study gave written informed consent.

### Statistical analysis

The statistical analysis was performed using the software Statistica 6.1. (InstallShield Software Corporation, USA) and SPSS 10.0 for Windows (SPSS Inc, USA). The results were presented as the median (Me) and the 25% and 75% quartiles Мe (Q1;Q3). The nonparametric tests were used to assess and analyze the obtained data: the Mann–Whitney U test or the Kolmogorov-Smirnov method (more than 50 cases in each group) were used to perform quantitative comparison of two independent groups, the Kruskal-Wallis test of variance by rank followed by the Mann–Whitney test with Bonferroni correction - for three independent groups comparison. The Spearman rank correlation coefficient was used to investigate the relationship between variables.

The stepwise logistic regression analysis with odds ratios (ORs) and 95% confidence interval (CI) were used to determine the prognostic significance of the parameters in the long-term prognosis. The Cox regression was used to evaluate the risk of unfavorable events; the impact of independent variables, predictors of the risk, was determined. A value of p < 0.05 was considered indicative of statistical significance.

## Results

Cardiovascular risk factor analysis showed that there was atherogenic dyslipidemia in all of the groups. At day 1 from MI onset, higher levels of TC, TAG, LDL, VLDL, and Apo B, a higher Apo B/Apo A1 ratio, and lower levels of antiatherogenic HDL and Apo A1 were observed in groups 1, 2 and 3 compared with the control group (Table 3).

**Table 3:** Parameters of lipid-transport function in the blood in patients with myocardial infarction on the 1st and 12th days of development of the disease

**Variables**	**Control, n = 33**	**Group 1, ≥ 75% stenosis of a coronary artery, n = 135**		**Group 2, ≥ 75% stenosis 2 coronary, n = 115**		**Group 3, ≥ 75% stenosis of 3 or more coronary arteries, n = 150**	
		**1-th day**	**12-th day**	**1-th day**	**12-th day**	**1-th day**	**12-th day**
TC, mmol/	4.30	5.91	5.80	5.68	5.66	5.83	5.59
	(3.65;5.50)	(5.56;6.65)^a^	(5.65;6.01)	(5.57;5,80)^a^	(5.42;5.87)	(5.74;5.99)^a^	(5.50;5.72)
TG, mmol/L	1.13	1.83	2.75	2.16	2.23	1.91	2.10
	(0.78;1.23)	(1.56;2.7)^a^	(2.52;3.1)^b^	(2.10;2.24)^a^	(2.14;2.37)	(1.82;2.02)^a^	(2.01;2.29)
HDL-C, mmol/L	1.31	1.12	1.01	0.95	1.19	0.98	0.96
	(1.02;1.72)	(0.96;1.25)^a^	(0.95;1.110)	(0.89;1,05)^ac^	(1.09;1.31)	(0.86;1,10)^ac^	(0.89;1.1)
LDL-C, mmol/L	2.03	2.73	3.30	2.32	3.36	2.96	3.38
	(1.51;2.55)	(2.59;2,99)	(3.23;3.41)^b^	(2.25;2.48)^a^	(3.27;356)^b^	(2.84;3.15)^a^	(3.27;3.57)^b^
VLDL-C, mmol/L	0.44	0.82	1.24	0.98	1.01	0.87	0.96
	(0.33;0.53)	(0.64;0.98)^a^	(1.15;1,36)^b^	(0.87:1.12)^a^	(0,96;1,15)	(0.78;0,97)^a^	(0,86;1.03)
Apo B, g/L	1.02	1.26	1.43	1.31	1.87	1.36	1.46
	(0.76;1.25)	(1.19;1.43)^a^	1.33;1,52)^b^	(1.23;1.1.43)^a^	(1,79;2,01)^b^	(1.26;1.45)^a^	(1.31;1.59)
Apo A1, g/L	1.43	1.30	1.56	1.25	1.65	1.24	1.33
	(1.29;1.73)	(1,21;1,42)	(1.50;1.68)	(1.19;1.32)^a^	(1.58;1,78)	(1.19;1,31)^a^	(1,29;1.49)
Аpо-В/Apо-А1	0.71	0.98	0.97	1.07	1.08	1.62	1.22
	(0.59;1.01)	(0.89;1.12)^a^	(0.88;1.11)	(1.01;1.19)^a^	(1.01;119)	(1,45;1,79)^a^	(1.12;1.35)
Atherogenic index	2.28	4.28	4.71	4.97	3.75	4.95	4,82
	(2.20;2.36)	(4.18;4,32)^a^	(4.62;4.76)	(4.85;5.10)^a^	(3.62;3,87)	(4.82;5,15)^a^	(4.69;4.96)
FFA, mmol/L	0.20	1.48	0.64	1.89	0.68	2.00	0.84
	(0.10;1.10)	(1.40;1.162)^a^	(0.58;0.69)^b^	(1.81;1.99)^ac^	(0.59;0.79)^b^	(1.91;2.15)^ac^	(0.78;0,98)^bc^

**Abbreviations:** TC. total cholesterol; TG. Triglycerides; HDL-C.cholesterol high-density lipoproteins; LDL-C. cholesterol low-density lipoproteins; VLDL-C. cholesterol very-low-density lipoproteins; Apo B. apolipoprotein B; Apo A1. apolipoprotein A.

**Note**: ^a^ - significant differences in the parameters with the control group, (p ≤ 0.05).

^b^ - significant difference parameters on day 1 and 12, (p ≤ 0.05).

^c^ - significant difference between 1 and 2, groups 1 and 3, (p ≤ 0.05).

Comparative analysis of the lipid profile in patients with different severities of CAD showed significant differences in HDL levels; HDL levels decreased with an increase in the number of affected arteries. HDL levels in groups 2 and 3 were 18% (р = 0.04) and 14% (р = 0.03) lower, respectively, than those in group 1.

By day 12 of the hospital stay, there was no significant improvement in the parameters under study. Moreover, TAG and VLDL (lipoproteins involved in TAG transport) levels in group 1 on day 12 were significantly higher than those on day 1. Atherogenic LDL levels were also significantly higher on day 12 in all of the groups compared with day 1 (23% [р = 0.04], 17% [р = 0.03], and 14% [р = 0.04], respectively).

Additionally, by the end of the hospital stay, patients in groups 1, 2 and 3 had higher Apo B (LDL) levels compared with controls. At day 12, antiatherogenic Apo A1 levels were significantly higher in group 2, but did not reach those of the control group. Notably, there was a trend towards a decrease in atherogenic TC levels in all of the groups, which suggests impairment of the early stage of recovery of metabolism because of the therapy.

**Figure 1:**
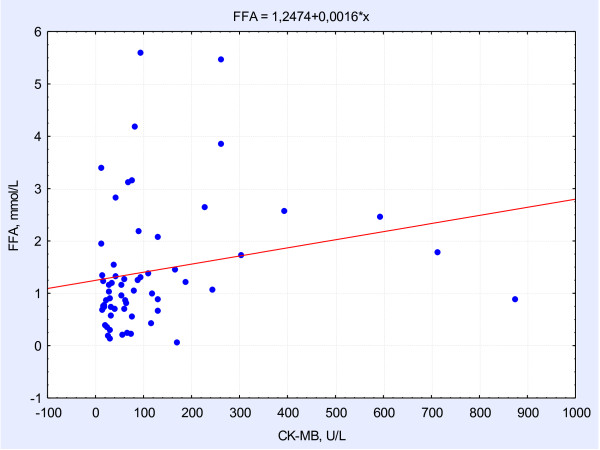
Correlations between CK-MB concentrations and FFA levels.

FFA concentrations on day 1 were, 7-fold, 9-fold, and 10-fold higher (р = 0.002, p = 0.01, p = 0.001) in groups 1, 2, and 3, respectively, compared with controls. At day 12, there was a 2.5–3-fold decrease in FFA levels in groups 1, 2 and 3, but they were still significantly higher compared with controls. At day 12, in groups 2 and 3 (multivessel CAD patients), FFA levels were higher than those in group 1.In MI patients, positive correlations were found between FFA levels at day 1 from MI onset and CK-MB activity, reflecting the size of myocardial necrotic focus (r = 0.401, р = 0.02) (Figure 1). Additionally, in MI patients, positive correlations were observed between FFA levels and ESV (r = 0.47, р = 0.01), and FFA levels and EDV (r = 0.53, р = 0.01), which indicated a strong association between increased FFA levels and postinfarct myocardial remodeling.

Oxidized LDL levels changed in all of the study groups. At day 1 from MI onset, oxidized LDL levels were significantly higher in groups 1, 2 and 3 than those in the control group. Oxidized LDL levels were significantly higher in groups 2 (d 1, 7%; р = 0.001) and 3 (d 1, 87%; р = 0.001) compared with group 1 at days 1 and 12 (Table 4). Notably, the increase in oxidized LDL levels was more pronounced in group 3 (increased by 75%) than in groups 1 and 2 (p = 0.001).

**Table 4:** Measurement of oxidation-modified LDL antibodies, oxidative status, and parameters of blood in patients with myocardial infarction on the 1st and 12th days of the disease

**Variables**	**Control, n = 33**	**Group 1, ≥ 75% stenosis of a coronary artery, n = 135**		**Group 2, ≥ 75% stenosis 2 coronary, n = 115**		**Group 3, ≥ 75% stenosis of 3 or more coronary arteries, n = 150**	
		**1-th day**	**12-th day**	**1-th day**	**12-th day**	**1-th day**	**12-th day**
Oxidized LDL, ng/mL	359.69 (324.56;389.23)	384.14 (349.52;412.1)	481.55 (411.25;533.21)^b^	409.13 (379.26;430.14)^ac^	605.23 (556.98;352.15)^bc^	717.16 (689.65;745.68)^acd^	1023.74 (956.21;1068.21)^bcd^
Oxidized LDL antibody, mU/mL	206.82 (189.63;225.32)	284.36 (251.23;312.23)^a^	395.72 (374.25;423.21)^b^	334.08 (316.59;354.12)^ac^	382.05 (369.24;399.96)^bc^	484.47 (459,82;509.51)^acd^	545.59 (521.32;574.25)^bcd^
Serum protein thiol, mcmol/L	515.1 (501.52;545.25)	646.42 (610.75;689.65)^a^	738.24 (698.23;769.6)^b^	735.84 (695.24;774.23)^a^	768.44 (726.24;789.65)	722.26 (692.12;761.15)^a^	753.34 (721.65;786.12)
Peroxide, mcmol/L	194.8 (189.32;208.45)	370.88 (345.26;396.21)^a^	451.05(423,89;478.21)	362.34 (326.25;386.25)^a^	408.85 (374.28;432.51)	391.00 (379.25;413.58)^a^	454.99 (423.11;489.25)

**Note: **^a^ - reliable differences in the parameters with the control group, (p ≤ 0.05).

^b^ - reliable difference parameters on day 1 and 12, (p ≤ 0.05).

^c^ - reliable difference between 1 and 2, groups 1 and 3, (p ≤ 0.05).

^d^ - reliable differences in the parameters between groups 2 and 3, (p ≤ 0.05)

By day 12, oxidized LDL levels were significantly increased in groups 1, 2 and 3 compared with day 1. The levels of oxidized LDL were 26% and 113% higher in groups 2 and 3, respectively, compared with group 1 (р = 0.001). Group 3 patients had 69% (р = 0.001) higher oxidized LDL levels at day 12 than group 2 patients.

A similar trend was observed for oxidized LDL antibody levels. Oxidized LDL antibody levels were higher in groups 1, 2 and 3 at day 1 compared with controls. In the subacute phase, oxidized LDL antibody levels increased by 37%, 61%, and 134% in groups 1, 2 and 3, respectively (р = 0.01, р = 0.005, р = 0.004, respectively).

Antibody levels increased with the number of affected arteries. On day 1, antibody levels were 17% (р = 0.003) and 70% (р = 0.002) lower in two-vessel and multivessel CAD patients, respectively, than in single-vessel CAD patients. More pronounced changes in antibody levels were observed in three-vessel CAD patients. On day 12, antibody levels in all of the groups started to increase and reached maximum levels in multivessel CAD patients.

On day 1, the level of thiol-containing compounds in single-vessel CAD patients was 25% (р = 0.004) higher than that in controls. In two- and multivessel CAD patients, thiol-containing compound levels were 43% and 40% higher, respectively, than those in controls (р = 0.007). At day 12, thiol-containing compound levels were similar to those on day 1 and were comparable among the groups with different disease severities. Serum peroxide levels showed the same tendency as thiol-containing compounds. Serum peroxide levels were higher at days 1 and 12 in groups 1, 2 and 3 compared with controls. There were no significant differences in serum peroxide levels among the groups.

We conducted a stepwise logistic regression analysis. All patients were divided into 2 groups: Group 1 - single-vessel disease, Group 2 multivessel disease, i.e. including two- and three-vessel disease (subdivided into Group 2 and Group 3).

The logistic regression analysis allowed identifying factors that have the closest relationship with multivessel disease (Table 5). Among the most statistically significant parameters were the following: oxidized LDL, their antibodies and FFA. An elevation of FFA on day 1 increased 2.6-fold the risk of IR development, on day 12–2.81-fold, oxidized LDL on day 1 increased 2.9 –fold, on day 12 – 1.82-fold, the antibodies to oxidized LDL on day 1–1.83-fold, on day 12–2.15-fold.

**Table 5:** Odds ratio (OR), 95% confidence interval (CI) and the area under the ROC-curve (AUC) in the development of multivessel СAD of myocardial infarction

**Variable**	**1-th day**				**12-th day**			
	**OR**	**95% CI**	**AUС**	**p-value**	**OR**	**95% CI**	**AUС**	**p-value**
TC, mmol/liter	1.14	1.01-1.02	0.51	0.41	0.93	0.99-1.05	0.47	0.64
TG, mmol/liter	1.31	0.75-2.31	0.58	0.12	2.10	0.99-4.48	0.52	0.06
HDL-C, mmol/liter	0.29	0.08-0.98	0.08	0.54	0.21	0.01-4.45	0.31	0.49
LDL-C, mmol/liter	0.98	0.98-0.99	0.13	0.68	0.74	0.40-1.35	0.32	0.67
VLDL-C, mmol/liter	0.99	0.47-3.60	0.47	0.96	0.51	0.80-1.23	0.34	0.02
apo B, g/L	1.20	0.17-4.40	0.61	0.43	0.82	0.61-1.60	0.52	0.54
apo A1, g/L	1.05	0.96-1.04	0.48	0.96	0.80	0.83-1.57	0.49	0.60
apo-В/apo-А1	0.98	1.52-9.21	0.65	0.90	1.83	0.17-2.49	0.51	0.06
Oxidized LDL	2.90	1.38-3.51	0.70	0.01	1.82	0.60-5.51	0.56	0.29
Oxidized LDL antibody	1.83	1.00-2.03	0.85	0.04	2.15	1.27-3.04	0.86	0.04
FFA, mcmol/L	2.60	1.30-3.09	0.82	0.04	2.81	1.99-3.25	0.82	0.03

р – reached the level of significance when compared with the control group.

## Discussion

The pathogenetic role of an imbalance in the lipid profile in cardiovascular diseases is well established. Large epidemiological studies have shown a correlation between blood levels of TC, LDL, and apoproteins and CAD mortality [10]. Dyslipidemia, which was found in our study on day 1, can be defined as higher atherogenic and lower antiatherogenic cholesterol fractions than controls. In the subacute MI phase, there is a tendency towards a decrease in cholesterol transport system markers, except for atherogenic LDL and Apo B; LDL and Apo B remain increased, leading to the need for early lipid-lowering therapy in this category of patients. However, we did not find any association between the severity of dyslipidemia and the extent of CAD. Therefore, there is a need to investigate other metabolic markers.

Recent studies have focused on identifying new biochemical markers of clinical complications of atherosclerosis, especially FFA [10]. FFA oxidation provides up to 70% of ATP to the heart, and other energy needs are satisfied by glucose oxidation. The intensity of FFA uptake by myocardial cells is determined by their plasma concentrations. Excess products and metabolites of FFA oxidation (acetyl-CoA, reduced NADPH, and FAD2) are natural inhibitors of pyruvate dehydrogenase complex enzymes, responsible for aerobic glucose oxidation, which leads to a decrease in myocardial glucose use [12]. In ischemia, the main metabolic pathway providing energy to cardiomyocytes is anerobic glycolysis because FFA oxidation requires more oxygen. In addition, altered myocardial use of FFAs due to myocardial ischemia and necrosis results in their accumulation in blood [13].The higher plasma FFA levels are, the lower their accumulation in tissue, and sometimes no glucose is released to cardiomyocytes. Excess FFA levels slow down not only glucose release, but also its use. This phenomenon is called the “glucose fatty acid cycle” (Randle) [14]. Therefore, high plasma levels of FFA decreases ATP production, which can lead to diastolic dysfunction, atrioventricular conduction delay, decreased fibrillation threshold, and ultimately, to heart failure. In the current study, analysis of FFA levels in MI patients showed that they were different in healthy subjects and in patients with a different number of affected coronary arteries, depending on the disease phase. Multivessel CAD in MI was associated with a more pronounced increase in FFA levels compared with controls, which can be regarded as a result of disturbance of metabolism and energy homeostasis in myocardial cells in this category of patients. In addition, the level of FFA can be regarded as a diagnostic indicator, reflecting the intensity of this derangement. However, high FFA levels in large-focal large MI reflects not only myocardial ischemia, but also the depth of myocardial necrosis. The results of correlation analysis support these suggestions.

Interestingly, a decrease in FFA levels in the early recovery phase was accompanied by an increase in TAG levels, which was more pronounced in single-vessel CAD patients than in multivessel disease patients, as well as an increase in VLDL levels in all of the groups. Previous studies have shown that this phenomenon is associated with development of insulin resistance under conditions of MI. This further decreases the activity of lipolytic enzymes and insulin-induced activation of TAG-producing enzymes in hepatocytes and adipocytes, as well as VLDL, which is responsible for the transport of these enzymes. Additionally, catecholamine levels in the recovery phase are decreased relative to the acute phase, and consequently, their stimulatory effect on lipolytic enzymes is less [6].

An increase in blood FFA and Apo B levels is known to reflect an increase in LDL, which is most susceptible to oxidation by active oxygen metabolites [15,16]. Oxidized LDL is thought to reflect excess production and accumulation of LDL in the blood. Studies have shown that oxidized LDL is strongly correlated with blood levels of fine dense LDL particles [17].

Oxidized LDL is cytotoxic for endothelial cells, which enhance adhesion of neutrophils, which causes endothelial injury [18]. Additionally, oxidized LDL contributes to activation of macrophages, induction of cyclooxygenase expression, and enhancement of proinflammatory prostaglandin (PGE2 and PHI2) and matrix metalloproteinase (MMP-2 and MMP-9) production, which are involved in erosion of fibrous plaque capsules.

As a result of oxidized LDL uptake by macrophages, foam cells are formed, which further accumulate in the endothelium and contribute to atherosclerotic plaque formation [18]. In our study, oxidized LDL levels in the blood were high during the entire hospital stay. Notably, the groups of patients significantly differed in oxidized LDL levels; the greater the number of affected vessels, the higher the oxidized LDL levels.

In the current study, interestingly, the levels of antibodies also depended on the severity of CAD, and the highest antibody levels were observed in multivessel CAD patients. The fact that there are antibodies to oxidized LDLs in healthy people is well-established, but their levels significantly increase if a patient has cardiovascular disease [11]. Currently, there is no consensus on the role of oxidized LDL antibodies. Some authors believe that the production of antibodies has a protective effect aimed at elimination of atherogenicoxidized LDLs. However, antibodies at increased levels form immune complexes with oxidized LDLs, which then bind to the intima and cause additional damage to the endothelium [17]. Regardless, most authors believe that antibodies to oxidized LDLs can potentially be atherogenic and their measurement can be used as part of risk assessment [11,19]. An increase in oxidized LDL antibody levels might be pathological, damaging the vessel wall, and eventually, lead to atherosclerotic changes.

One of the causes of accumulation of oxidized LDL and its antibodies in the blood is thought to be impaired pro- and antioxidant systems. Our study showed an increase in the levels of peroxides and thiol-containing compounds, reflecting the oxidative status, and representing the pro- and antioxidant systems, respectively. However, an increase in these markers does not depend on the severity of CAD. This suggests that this process is non-specific, and imbalance in pro/antioxidant systems can be regarded as a general pathological response in MI.

## Conclusion

Thus, FFA, LDL and antibodies appear to be the most informative indices reflecting the severity of atherosclerotic coronary lesions that retain high concentrations throughout the in-hospital period and, moreover, these markers are the most elevated in multivessel disease.

## Abbreviations

MI: Myocardial infarction; CAD: Coronary artery disease; FFA: Free fatty acids; ACS: Acute coronary syndrome; TC: Total cholesterol; TG: Triglycerides; HDL-C: Cholesterol high-density lipoproteins; LDL-C: Cholesterol low-density lipoproteins; VLDL-C: Cholesterol very-low-density lipoproteins; Apo B: Apolipoprotein B; Apo A1: Apolipoprotein A.

## Competing interests

This manuscript has been read and approved by all the authors. This paper is unique and is not under consideration by any other journal and has not been published elsewhere. The authors of this paper report no conflicts of interest. The authors confirm that they have permission to reproduce any copyrighted material.

## Authors’ contributions

OG was principal investigator, study coordinator and investigator, participated in all stages of recruitment of the patients and in analysis of the data, drafted and reviewed critically the manuscript. EU,VK was study coordinator and investigator, participated in all stages of recruitment of the patients and in analysis of the data, drafted and reviewed critically the manuscript; EB and YD, AS was study investigator, participated in all stages of recruitment of patients and reviewed critically the manuscript. OB was principal investigator. All other study investigators conducted the study and collected the data. All authors read and approved the final manuscript.
